# Developing a machine learning model to assist in predicting treatment success in children with drug-resistant epilepsy

**DOI:** 10.3389/fneur.2025.1701755

**Published:** 2025-12-03

**Authors:** Achmad Rafli, Wisnu Ananta Kusuma, Setyo Handryastuti, Irawan Mangunatmadja, Rahmad Mulyadi, Aria Kekalih, Anggi Gayatri, Elisabeth Herini

**Affiliations:** 1 Doctoral Program in Medical Sciences, Faculty of Medicine Universitas Indonesia, Jakarta, Indonesia; 2 Department of Child Health, Faculty of Medicine Universitas Indonesia, Cipto Mangunkusumo Tertiary General Hospital, Jakarta, Indonesia; 3 Bioinformatics Study Program, Faculty of Mathematics and Natural Sciences, IPB University, Bogor, Indonesia; 4 School of Data Science, Mathematics, and Informatics, IPB University, Bogor, Indonesia; 5 Department of Radiology, Faculty of Medicine Universitas Indonesia, Cipto Mangunkusumo Tertiary General Hospital, Jakarta, Indonesia; 6 Department of Community Medicine, Faculty of Medicine Universitas Indonesia, Jakarta, Indonesia; 7 Department of Pharmacology and Therapeutic, Faculty of Medicine Universitas Indonesia, Jakarta, Indonesia; 8 Department of Child Health, Faculty of Medicine, Public Health and Nursing Universitas Gadjah Mada, Yogyakarta, Indonesia

**Keywords:** children, drug-resistant epilepsy, antiepileptic drug, machine learning, preliminary study

## Abstract

Currently, the successfulness of reducing seizures through the selection of appropriate antiepileptic drugs (AED) in children with drug-resistant epilepsy remains a challenge due to variability characteristic in patients. This study aims to develop and evaluate machine learning models to treatment success in pediatric patients with drug-resistant epilepsy. This study will be conducted with an ambispective cohort. A total of 215 subjects will be taken from patients in Cipto Mangunkusumo Referral Hospital and Harapan Kita Child and Mother Hospital Jakarta, Indonesia. Supporting examinations will be also performed such as electroencephalography (EEG) and modified HARNESS Magnetic Resonance Imaging (MRI). The collected data will be analyzed by machine learning with several algorithms including support vector machine (SVM), decision tree (DT), random forest (RF), gradient boosting (GB), and their performance will be compared to determine the best model. This is the first study to utilize machine learning by integrating clinical data, EEG, MRI, and medication history to predict treatment success in pediatric patients with drug-resistant epilepsy in Indonesia. The developed model is expected to serve as a clinical decision supporting tool for pediatric neurologists to predict seizure control in children with DRE and determine appropriate therapeutic adjustments with more aggressively when uncontrolled seizures are predicted.

## 1 Introduction

Epilepsy is one of the most common neurological disorders and is characterized by recurrent uprovoked seizures (1–3). According to the ILAE 2010 definition, drug-resistant epilepsy is a condition characterized by seizure control failure with the use of at least two AEDs at maximum doses (4). About 20–40% of children with epilepsy can develop drug-resistant seizures despite appropriate AED (5). According to the latest medical records in 2020–2024 at the child neurology polyclinic of Cipto Mangunkusumo Hospital, the number of epilepsy patients visit was 5,760 (40%) of the total outpatient data 14,402 patients. Approximately, there are around 200 children with drug-resistant epilepsy which also continues to rise (6).

Several important things in selecting AED for children with drug-resistant epilepsy are seizure type, drug mechanism of action, side effects, and minimal interactions (7, 8). There are limitations in selecting appropriate combinations of AED due to the variability characteristics in patients, so that each individual undergoes a different response in treatment. Currently in Indonesia, AED is given to patients based on the type of seizure or epilepsy approach. Precise and accurate treatment is challenging for patients with drug-resistant epilepsy. One approach that can be used to predict the successful treatment is using artificial intelligence (AI).

The use of AI has grown rapidly in medicine, especially in epilepsy (9–13). Machine learning (ML) is a part of AI which utilizes learning features to build systems that can learn and improve their performance based on the provided data (14, 15). The use of ML in the field of epilepsy has great potential and has been used to predict outcomes in several studies (9). This approach can integrate various patient’s data and analysis results into one platform. As of now, in Indonesia, there is still no use of AI to predict treatment success in pediatric patients with drug-resistant epilepsy. This study aims to develop machine learning models to predict treatment success in pediatric drug-resistant epilepsy, which may assist pediatric neurologists in making clinical decisions regarding patient’s seizure control status.

## 2 Methods

### 2.1 Study design

This protocol will be conducted in ambispective cohort and multicentre study, involving 215 children patients from Cipto Mangunkusumo Hospital and Harapan Kita Hospital. Subject data will be collected from electronic medical records, examinations, interviews, EEG and brain MRI examination in children with drug-resistant epilepsy from January 2020 to August 2025. The subjects will undergo follow-up in an outpatient clinic over 3 months period.

### 2.2 Predictors

Several features data that will be the outcome predictors in model analysis are listed in Table 1.

**Table 1:** Predictors features.

Predictors features	Categories
Age of epilepsy diagnosis	<1 years
	1–< 5 years
	5–< 10 years
	10–< 15 years
	15–18 years
Type of epilepsy	Focal
	Generalized
	Focal to Generalized
	Epilepsy syndrome
EEG results	Normal
	Abnormal without epileptiform wave
	Abnormal with epileptiform wave
	Epilepsy syndrome
MRI results	Normal
	Abnormal epileptogenic
	Abnormal non-epileptogenic
AED combination	Valproic acid and levetiracetam
	Valproic acid and topiramate
	Valproic acid and carbamazepine or oxcarbazepine
	Valproic acid and levetiracetam and topiramate
	Valproic acid and levetiracetam and clobazam

### 2.3 Outcomes

The outcome of this study is to develop an initial supervised learning ML model for predicting treatment success in pediatric patients with drug-resistant epilepsy based on patient characteristics. This study used a 3 months interval after the maximum dose of AED was administered to predict treatment success and ≥75% seizure reduction as a major response (16, 17). Treatment success in patients is categorised as controlled if there is a reduction in seizure frequency ≥75% compared to baseline after 3 months of treatment and uncontrolled if there is a reduction in seizure frequency <75% compared to baseline after 3 months of treatment. This machine learning model is expected to help paediatric neurologists predict treatment success in patients with drug-resistant epilepsy and used in a resource-limited setting.

### 2.4 Participants

This protocol will be used in children who are regular patients from pediatric neurology clinics of Cipto Mangunkusumo Referral Hospital and Harapan Kita Child and Mother Hospital Jakarta, Indonesia with diagnosis drug-resistant epilepsy based on the established guideline (4). Patients who are eligible for this study must fill in an informed consent form before the study begins.

#### 2.4.1 Eligibility criteria

Inclusion criteria include children aged 1 month to 18 years with confirmed diagnosis of drug-resistant epilepsy, subject currently receiving two or more of the following AED combinations:

- Valproic acid and levetiracetam
- Valproic acid and topiramate
- Valproic acid and carbamazepine or oxcarbazepine
- Valproic acid and levetiracetam and topiramate
- Valproic acid and levetiracetam and clobazam

Exclusion criteria are subjects with drug-resistant epilepsy etiology due to structural factors such as tumours, vascular disorders, and metabolic abnormalities. Subjects with uncontrolled seizures caused by AED pseudoresistance factors are also excluded, such as AED dosage is not optimal, not taking AED regularly as prescribed, and sleep deprivation.

### 2.5 Sample size estimation

The sample estimation use the rule of thumb method. According to the rule of thumb method, sample is considered sufficient if the number of outcome cases is approximately 10 times the number of predictors being studied, and prevalence of the cases. In this study, five variables are used as predictors. The prevalence of uncontrolled seizures in drug-resistant epilepsy patients is 67% (12). The proportion of uncontrolled seizure status during the 3-month monitoring period in drug-resistant epilepsy patients currently still lacks of studies. Based on this, the required minimum sample size is 10 × 5/0.67 = 74 patients.

### 2.6 Study procedure

#### 2.6.1 Data collection

Data will be collected through parents or caregivers interview, electronic medical record, EEG and MRI results. The subject will also be scheduled for EEG and brain MRI examinations with modified HARNESS MRI. Subject’s characteristic data will then be displayed in Table 2.

**Table 2:** Dummy table subject’s characteristics.

Subject’s characteristics	*n* (%)
Gender	
Male	
Female	
Age	
<1 years	
1– < 5 years	
5– < 10 years	
10– < 15 years	
15–18 years	
Age of epilepsy diagnosis	
<1 years	
1– < 5 years	
5– < 10 years	
10– < 15 years	
15–18 years	
Number of AED used	
2 AED	
3 AED	
AED combination	
Valproic acid + Levetiracetam	
Valproic acid + Topiramat	
Valproic acid + Carbamazepin/Okscarbazepin	
Valproic acid + Levetiracetam + Topiramat	
Valproic acid + Levetiracetam + Clobazam	
Type of epilepsy	
Focal	
Generalized	
Focal to generalized	
Epilepsy syndrome	
EEG results	
Normal	
Abnormal without epileptiform wave	
Abnormal with epileptiform wave	
Epilepsy syndrome	
MRI results	
Normal	
Abnormal epileptogenic	
Abnormal non-epileptogenic	
Seizure control status in 3 months	
Controlled	
Uncontrolled	

**Figure 1:**
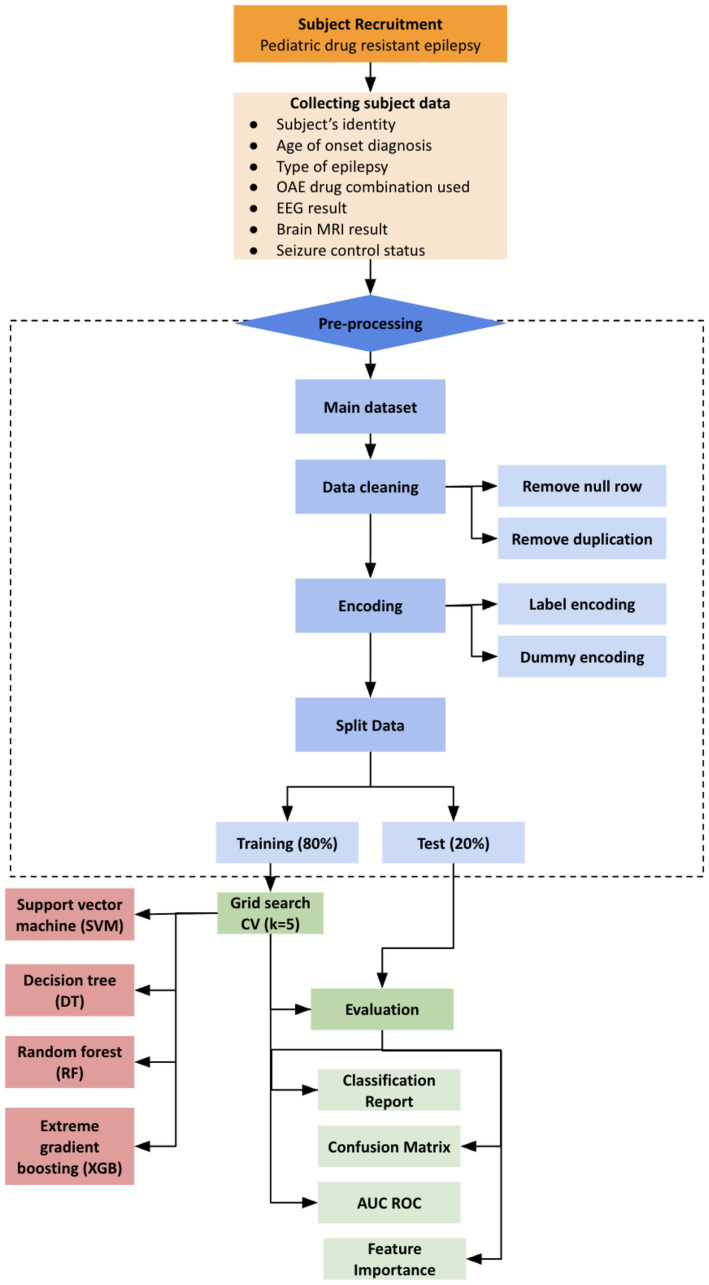
Study procedure.

After collecting all the subject’s data, the dataset will undergo pre-processing and processing for developing the machine learning models. This procedure will be clearly explained in Figure 1.

#### 2.6.2 EEG examination

EEG examination is performed at the EEG Unit of Cipto Mangunkusumo Referral Hospital and Harapan Kita Child and Mother Hospital Jakarta, Indonesia with standard pediatric EEG procedure using Caldwell and Neosoft machines. If the subject has undergone EEG examination in the last 3 months, repetition is not required. The EEG result will be interpreted by two paediatric neurologists who are certified in international EEG and will be assessed by kappa test. EEG results are categorized into normal, abnormal non-epileptiform, abnormal with epileptiform or epilepsy syndrome (see Tables 3, 4).

**Table 3:** Dummy table for machine learning evaluation in data training.

Algorithm	ACC	AUC	Precision (PPV)	F1-score	Recall (sensitivity)
Support vector machine (SVM)					
Decision tree (DT)					
Random forest (RF)					
Extreme gradient boosting (XGB)					

**Table 4:** Dummy table for machine learning evaluation in data testing.

Algorithm	ACC	AUC	Precision (PPV)	F1-score	Recall (sensitivity)
Support vector machine (SVM)					
Decision tree (DT)					
Random forest (RF)					
Extreme gradient boosting (XGB)					

##### 2.6.2.1 Brain MRI examination

Brain MRI examination is performed at the radiology unit of Cipto Mangunkusumo Referral Hospital and Harapan Kita Child and Mother Hospital Jakarta, Indonesia using Philips, GE and Siemens machines. This brain MRI examination will use a modified Harmonized Neuroimaging of Epilepsy Structural Sequences (HARNESS-MRI) epilepsy protocol for children including: T1-weighted 3D, T2-weighted coronal, T2-weighted coronal hippocampus, T2-weighted axial hippocampus, Diffusion-weighted Imaging (DWI), Derived apparent diffusion coefficient (dADC), Susceptibility-weighted imaging (SWI), Venous spin weighted imaging (VSWI), Fluid attenuation inversion recovery (FLAIR) coronal hippocampus, Fluid attenuation inversion recovery (FLAIR) axial hippocampus, Short tau inversion recovery (STIR) coronal hippocampus, and Short tau inversion recovery (STIR) axial hippocampus. The aim of using modified HARNESS-MRI is to see a better lesion with several additional sequences apart from the major HARNESS sequences. If the subject has previously undergone brain MRI examination in the last 6 months, repetition is not required. This result will be interpreted by one consultant neuroradiologist. Brain MRI results are categorized into normal, abnormal non-epileptogenic, or abnormal with epileptogenic.

### 2.7 Machine learning development

The initial dataset is first collected using Microsoft Excel and then later imported into the Yavai program which use Python language program. The dataset will then undergo data cleaning, which includes removing data entries with empty values and duplication. Afterwards, the data will be encoded into numerical form to make it readable by machine learning programs. Feature selection will not be performed in this study, as the researcher intends to use all available data features. The data will be randomly divided into 80% training and 20% testing data. Hyperparameter tuning on training data will be conducted using the grid search method with a 5-fold cross-validation procedure on each model. The data will be divided into five subsets in this procedure, with four subsets used as the training set and one subset used for validation. This process will be repeated five times to assess the performance of each model during training and determine the best parameters. Several algorithms will be applied in this study, including support vector machine (SVM), decision tree (DT), random forest (RF), gradient boosting (GB), and extreme gradient boosting (XGBoost). Each machine learning model performance will be evaluated using a classification report, confusion matrix, and AUC-ROC value. The classification report evaluation includes precision, recall, and F1-score. Meanwhile, the confusion matrix will be presented in table figure, including TP (True Positive), TN (True Negative), FP (False Positive), and FN (False Negative) between the predicted values in machine learning and the actual values. The AUC-ROC curve evaluation is used to measure the discriminatory ability of a model.

## 3 Discussion

Drug-resistant epilepsy is caused by multifactorial etiology, including type of seizure, onset of seizure, family history of seizure, abnormal result of EEG and MRI, abnormal neurology examination and history of NICU admission (5, 18–22). Pharmacokinetic and pharmacodynamic mechanisms are related to the pathophysiology of drug-resistant epilepsy. The pharmacokinetic mechanisms involved in drug resistance, including limitations in achieving optimal antiepileptic drug (AED) concentrations at the site of action which is influenced by several factors such as solubility in blood, absorption, metabolism, and drug elimination. Meanwhile, pharmacodynamic mechanisms relate to factors that change AEDs effects at their site of action, such as at synapses, ion channels, and receptors (23, 24).

In 2019, ILAE recommended the HARNESS-MRI protocol as the optimal imaging protocol for epilepsy (25). The use of the epilepsy protocol in MRI can improve the lesion confirmation success rate from 49 to 72% (26). HARNESS-MRI protocol consists of three basic sequences that can be applied to both adult and pediatric patients (25). Therefore, this study will also perform a similar protocol with the HARNESS-MRI protocol, which is expected to be more sensitive and specific in identifying epilepsy focus with some additional sequences.

The treatment goal for drug-resistant epilepsy is to achieve reduction in seizure frequency and to improve patient’s quality of life with minimal side effects from AED (27). Treatment for drug-resistant epilepsy still faces numerous challenges, particularly in optimizing medication strategies (28). In administering AED for drug-resistant epilepsy, several aspects need to be considered, including ensuring the absence of pseudo-resistance, selecting appropriate AED combination, and considering non-pharmacological treatment options (29). The selection of AED combination therapy should be based on the diagnosis, mechanisms of action and potential side effects. Currently, AED combinations are generally chosen based on clinical experience using a “trial and error” approach (30, 31). Several studies have summarized the use of AED combinations that have proven effective in controlling epilepsy (32, 33). In this study, OAE combination regimens were also included as one of the variables, which consist of the five most commonly used OAE combination regimens. These combinations are selected based on the types of first-line and second-line drugs and are also generally covered under Indonesia’s national health insurance (34).

ML has been used in several tasks, including diagnosis, treatment, detection, and outcome prediction in healthcare, especially in epilepsy (9). Several studies on epilepsy in children have used machine learning to predict the success of therapy. Some of the algorithms that are commonly used are decision tree, random forest, support vector machine, gradient boosting, and others which are similar to those used in this study (10–12). The study by Wu et al. (11) in predicting the success of AED treatment in patients with familial genetic generalized epilepsy (GGE) showed that random forest was the best model compared to 13 other models. Another study by Devinsky et al. (35) used random forest algorithm to predict the success of therapy in epilepsy patients, while Zhang et al. (13) used SVM algorithm to predict the success of levetiracetam therapy in epilepsy patients.

This study has limitations in terms of its relatively small sample size and therefore further studies are expected to have larger sample sizes. Prior to this time, there have been no studies in Indonesia that have used machine learning to predict the success of AED therapy by integrating patient data, including clinical data, EEG, MRI, and medication history. This model is expected to serve as a prototype that can later be developed and assist pediatric neurologists in predicting the success of therapy and determining the therapy or intervention to be done further for the patient.
